# Clinical evaluation of metagenomic next-generation sequencing in unbiased pathogen diagnosis of urinary tract infection

**DOI:** 10.1186/s12967-023-04562-0

**Published:** 2023-10-27

**Authors:** Ye Wang, Ting Chen, Shengwei Zhang, Lei Zhang, Qian Li, Qingyu Lv, Decong Kong, Hua Jiang, Yuhao Ren, Yongqiang Jiang, Yan Li, Wenhua Huang, Peng Liu

**Affiliations:** 1 State Key Laboratory of Pathogen and Biosecurity, Beijing Institute of Microbiology and Epidemiology, Academy of Military Medical Sciences, Beijing, China; 2 Department of Critical Care Medicine, The Fifth Medical Center of PLA General Hospital, Beijing, China

**Keywords:** Metagenomic next-generation sequencing (mNGS), Pathogen diagnosis, Urinary tract infections (UTIs), MinION, Automatic bioinformatics

## Abstract

**Background:**

Early availability of pathogen identification in urinary tract infections (UTIs) has critical importance in disease management. Metagenomic next-generation sequencing (mNGS) has the potential to transform how acute and serious infections are diagnosed by offering unbiased and culture-free pathogen detection. However, clinical experience with application of the mNGS test is relatively limited.

**Methods:**

We therefore established a MinION-based mNGS pathogens diagnostic platform and evaluated its potential for clinical implementation in UTIs with clinical samples. 213 urine samples from patients with suspected UTIs were included and subjected to mNGS testing using the MinION platform. mNGS results were compared to the gold standard of clinical culture and composite standard of combining clinical testing, confirmatory qPCR testing, and clinical adjudication by doctors.

**Results:**

The mNGS exhibited a sensitivity of 81.4% and a specificity of 92.3%, along with a positive predictive value of 96.6%, a negative predictive value of 64.9%, and an overall accuracy of 84.4%, all of which were determined based on the gold standard of routine culture results. When assessed against the composite standard, the sensitivity and specificity both increased to 89.9% and 100%, respectively, while the accuracy rose to 92.4%. Notably, the positive predictive value and negative predictive value also saw improvements, reaching 100% and 76.8%, respectively. Moreover, this diagnostic platform successfully identified dsDNA viruses. Among the 65 culture-negative samples, the viral detection rate reached 33.8% (22/65) and was subsequently validated through qPCR. Furthermore, the automatic bioinformatics pipeline we developed enabled one-click analysis from data to results, leading to a significant reduction in diagnosis time.

**Conclusion:**

These results demonstrate that the pathogen detection performance of mNGS is sufficient for diagnostic testing in clinical settings. As the method is generally unbiased, it can improve diagnostic testing of UTIs and other microbial infections.

**Supplementary Information:**

The online version contains supplementary material available at 10.1186/s12967-023-04562-0.

## Background

Infectious diseases remain the leading cause of morbidity and mortality in all patient populations worldwide [1]. Among these, urinary tract infections (UTIs) are one of the most prevalent, affecting more than 150 million people annually [2]. Patients with UTIs are frequently among those who are immunocompromised due to preexisting conditions such as cancer, hereditary syndromes, or transplantation, especially if they are in tertiary care medical centers which makes them extremely vulnerable to infections. In these settings, the causal agent of UTIs can include a number of both common and uncommon pathogens, ranging from viruses to bacteria, fungi, and parasites [3]. A rapid detection method for the causative pathogen(s) enables early targeted antimicrobial therapy, which significantly increases a patient’s survival rate, prevents subsequent complications, and reduces drug-related side effects as well as medical expenses [4].

A comprehensive identification of multiple kinds of pathogens has always been a difficult but critical issue for infectious disease clinicians. Conventional in vitro culture methodology is the primary method for the detection of fungal and bacterial pathogens in clinical laboratories. However, this technique has a long turnaround time and remains limited to the detection of relatively few culturable organisms [5, 6].

Standard molecular methods such as PCR-based and targeted sequencing strategies (e.g., 16S ribosomal RNA (rRNA) sequencing for bacteria/archaea and internal transcribed spacer (ITS) sequencing for fungi) require preliminary knowledge, which can sometimes be impractical due to the complicated pathogen spectrum [7]. In addition, these methods are only effective for the detection of a single type of pathogen at a time, and may require multiple assays for broad spectrum detection. Current diagnostics for pathogen identification mainly depend on culture- and molecular-based approaches, which are inadequate in regards to specificity, sensitivity, and time to diagnosis [8].

Metagenomic next-generation sequencing (mNGS) is advantageous because of its broad range identification capacity and can address some of the drawbacks and limitations of culture and molecular-based methods described above. mNGS is a hypothesis-free and largely unbiased approach that has the potential to detect all known as well as unexpected pathogens, and can even lead to the discovery of new organisms in a clinical sample. It is especially suitable for rare, novel, and atypical etiologies of complicated infectious diseases, as well as the molecular diagnosis of polymicrobial infections [3, 9, 10].

There are a variety of high-throughput sequencing technologies that are commercially available as commercial platforms, with Illumina’s sequencing-by-synthesis as the dominant contender. However, this process requires > 18 h from sample to result, which is suboptimal for the rapid etiological diagnosis of acute infections [11]. MinION (Oxford Nanopore Technologies, UK) is a representative of recently developed sequencing platforms: a handheld sequencer that can immediately process generated reads in real time, has the greater potential to perform point-of-care testing (POCT), and reduces time of diagnosis down to a few hours [4]. The potential of this sequencing platform, with respect to clinical microbiology, has already been shown in several studies ranging from outbreak surveillances to infection diagnostics from various clinical samples (urine, blood, cerebrospinal fluid, implants, feces, sputum) and antimicrobial resistance profiling [12–17]. However, to date the lack of practical mNGS adoption for clinical microbiology can be attributed to insufficient case cohorts for the clinical validation of mNGS, difficulty in discriminating pathogens from colonizers or contaminants, and the lack of bioinformatics software tailored for clinical diagnostic use [3, 18, 19].

In this study, a MinION-based mNGS pathogen identification platform was established and evaluated its potential for the clinical implementation of rapid pathogen diagnosis in urinary tract infection. By developing an automatic data processing pipeline, the bioinformatic processing was simplified and the whole diagnosis time by mNGS finally reduced from several days to four hours.

## Materials and methods

### Study design and ethics

**Fig. 1:**
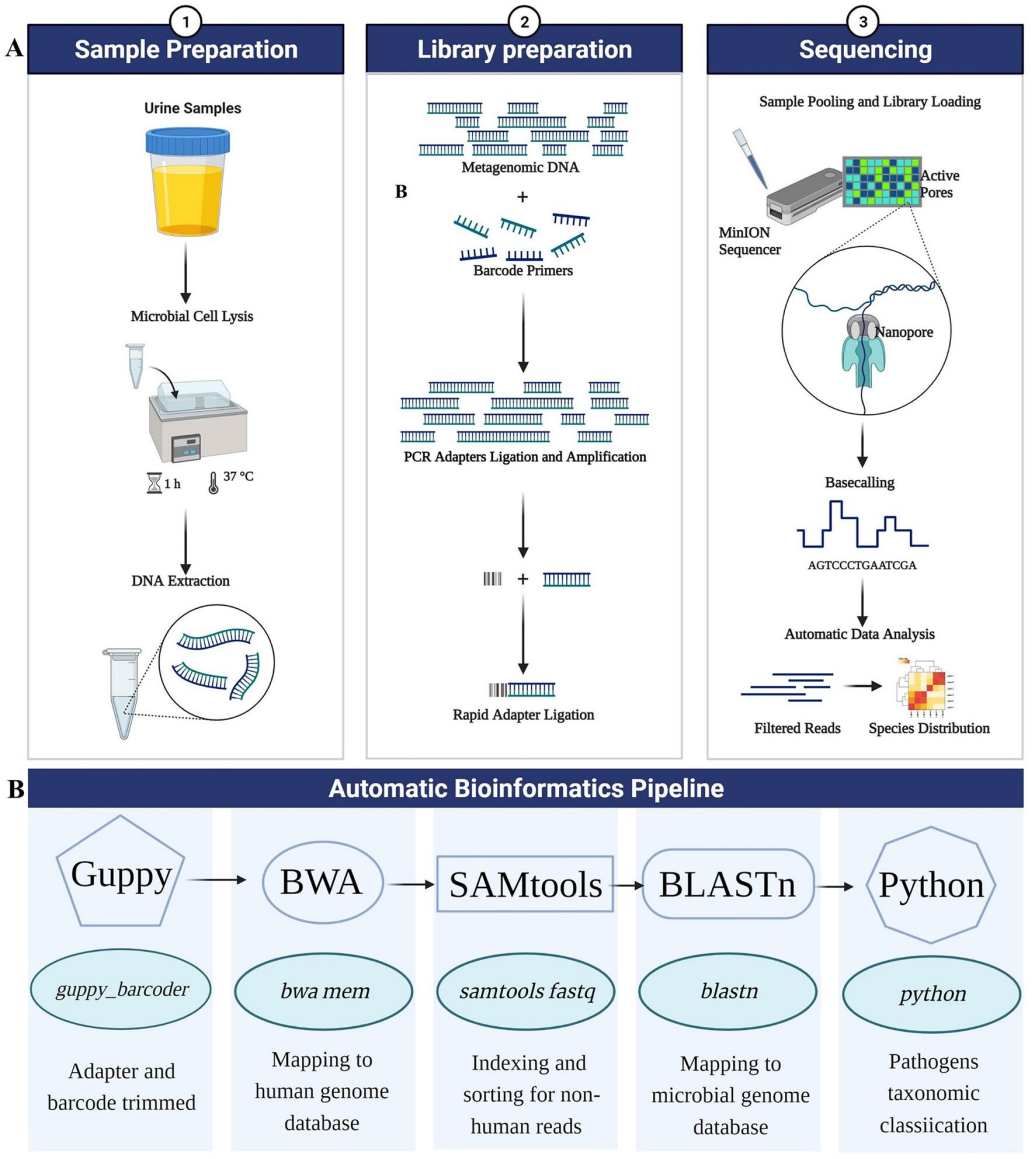
Schematic of the MinION-based mNGS workflow. **A** Schematic of the complete mNGS testing from sample reception to diagnosis results. **B** Detailed workflow of the automatic bioinformation pipeline, APDNS

A cohort of 213 cases was collected from consecutively hospitalized and emergency suspected UTI patients from Dongfang Hospital in Beijing, China and the Fifth Medical Center of PLA General Hospital between December 2020, and December 2021 to perform mNGS testing and assess the performance of this method in real-life clinical practices. 148 samples with culture-positive results and 65 samples with culture-negative results were used as test subjects. All cases were followed the inclusion criteria of symptoms including urinary urgency, frequent urination, and painful urination. Additional 39 urine samples from healthy individuals were collected to aid in building the positive detection criteria of mNGS and assessing the performance of the mNGS workflow. We previously examined a subset (N = 76) of the current 213 samples, in which we focused exclusively on bacterial pathogens, including detection of causative bacteria and known antimicrobial resistance genes (ARGs) [2]. In the present study, we have added 137 new samples and have conducted analysis of bacterial, fungal, and viral pathogens with a newly developed automatic bioinformatics pipeline (*Automatic pathogen diagnosis by nanopore sequencing, APDNS*). The workflow for etiological diagnosis of UTI by MinION-based mNGS testing is shown in Fig. 1.

### Sample collection and processing

Clinical microbiological analysis of the 213 urine samples was performed according to standards formulated by the Clinical and Laboratory Standards Institute (CLSI). Briefly, 10μL of urine were inoculated onto blood agar plates (bioMérieux) and incubated for 16–24 h at 35 °C ± 2 °C in aerobic conditions. If cultures were negative for colony formation, plates were incubated continually until 48 h. Bacterial and fungal counts more than 10^2^ CFU were regarded as positive. The remnants after routine clinical microbiological laboratory testing were frozen at -80℃ until mNGS analysis. Data on the demographic characteristics and clinical laboratory findings of the 213 patients were extracted from the patients’ medical records. The final diagnosis of UTIs was based on microbiological tests, microscopy, and clinical adjudications.

### DNA extraction

One aliquot of 1 mL urine sample was taken into 1.5 mL Eppendorf tube and centrifuged at 20000 × *g* for 5 min to enrich for pathogens. The resulting pellet was resuspended in 200 μL remanent supernatant with brief, gentle vortexing. Then, 5 μL of lytic enzyme solution (Qiagen Inc., Hilden, Germany) and 10 µL of MetaPolyzyme (Sigma Aldrich, Darmstadt, Germany; reconstituted in 750µL PBS) were added to the samples and mixed by pipetting. Mixed samples were incubated at 37 °C for 1 h to lyse microbial cells. DNA was extracted from each sample post-lysis using the IndiSpin Pathogen Kit (Indical Bioscience GmbH, Leipzig, Germany).

Sterile deionized water was extracted alongside the specimens as a negative control (NTC). The concentrations of DNA were measured using Qubit 4.0 fluorometer (Thermo Fisher Scientific, USA) with a dsDNA HS Assay kit.

### Library preparation and sequencing

The samples included in this dataset were processed as described above and sequenced regardless of how much the DNA concentration were, to provide an accurate representation of the data that would likely be obtained from metagenomic analysis of urine in clinical settings.

Library preparation was performed using the PCR Barcoding Kit (SQK-PBK004, Oxford Nanopore Technologies, UK) according to the manufacturer’s protocols, with 2 min extension and 15 cycles in the PCR amplification step. Up to six barcoded samples were loaded per flow cell for each sequencing run, along with an NTC sample to allow for the surveillance of possible contamination. All NTCs underwent the same wet lab procedure and bioinformatic analysis as the clinical samples.

Nanopore sequencing was performed using R9.4.1 flow cells (FLO-MIN106, Oxford Nanopore Technologies, UK) on MinION instrument. A total of 75µL of library DNA was loaded into the flow cell according to the manufacturer’s instructions. ONT MinKNOW GUI software (version 4.2.8) was used to collect raw sequencing data and the sequencing run was continued for 1–2 h to collect approximately 60 k reads per sample. Between each run, the flow cell was washed using Flow Cell Wash Kit (WXP-WSH004, Oxford Nanopore Technologies, UK) according to the manufacturer’s protocols. Sample treatment and library preparation pipeline has been described in a previous study conducted by this laboratory [2].

### Bioinformatics analysis

For the ease and speed of processing raw sequencing data, an automatic bioinformatics pipeline was developed that comprised of a series of Linux shell and python scripts. It incorporates several open-source tools, including ont-Guppy (version 6.0.1), bwa (version 0.7.17), BLASTn (version 2.10.1), SeqKit (version 2.1.0) and SAMtools (version 1.7). The Refseq database was downloaded from National Center Biotechnology Information (release version 205; ftp://ftp.ncbi.nlm.nih.gov/genomes/refseq) and used for species classification. The database contained 238,362 bacterial and 8265 viral genomes, 429 fungal and 96 protozoal genomes associated with human diseases. The automatic bioinformatics pipeline accepted raw fq.gz file as inputs and analyzes them on an Ubuntu 18.10 computational sever. The processing step consisted of (1) trimming adapters using Guppy, (2) subtracting human host sequences mapped to the human reference genome (GRCh38) using Burrows-Wheeler alignment with BWA-MEM algorithm, (3) processing the SAM file output in previous step with SAMtools to generate non-human reads, (4) classifying all non-human reads by simultaneous alignment to microbial genome databases consisting of viruses, bacteria, fungi, and parasites using BLASTn, and finally (5) two custom Python scripts were used to perform taxonomic classification including extract the mapped species names and calculate the mapped reads number of each detected species; further, species detected were sorted and listed with some extra information including reads number, proportion and so on from high to low in csv format. The detailed steps and parameters are shown in Fig. 1.

This pipeline allows its user to customize the reference database according to necessity. For example, for 16S rRNA sequencing, the Refseq bacterial database or specific 16S database can be selected to save alignment time. For metagenomic sequencing, the Refseq microbial database or the more comprehensive NT database can be selected to cover as many potential pathogens as possible. This automatic bioinformatics pipeline is available at https://github.com/gitzl222/APDNS/.

### Criteria for positive mNGS results

To minimize false-positive results from the low-level DNA background of the reagents, microbial contamination, and urethral colonizing flora, threshold criteria were established for pathogen detection. Then, mNGS analytical performance measures were assessed based on those criteria. A total of 117 urine samples (78 culture-positives and 39 culture-negatives) were selected randomly as the training dataset to determine positive thresholds based on sequencing data, and the remaining samples were divided into the validation dataset. For the identification of bacterial and fungal pathogens, a nRPM (normalized reads per million, nRPM = 100 RPM) was defined as the normalized percentage of target pathogen reads in the remaining total reads after barcode trimming, referring to the threshold defined by Chiu et al. [20]. In order to determine the optimal threshold value and maximize the accuracy of pathogen detection, receiver operator characteristic (ROC) curves were plotted for the training dataset at varying nRPM values and read numbers of target pathogen, the nRPM threshold and reads number threshold were determined at the most optimal Youden’s Index. The ROC curve was plotted using GraphPad prism 8 software. Positive criteria for fungal and bacterial pathogens detection were set by meeting the following conditions: (1) be above the optimal nRPM threshold, (2) be above the lowest-read number threshold of target pathogen, and (3) exclude the non-pathogenic species or probiotics (e.g., *Lactobacillus crispatus* and *Lactobacillus iners)* which have been reported in other published [21, 22].

For identification of viruses, positive detection was defined as 1 or more reads with > 80% identity mapped [19].

### Real-time quantitative polymerase chain reaction

For the confirmation of positive pathogen results from mNGS, qPCR testing was performed using StepOnePlus™ Real-Time PCR System (Life Technologies, Darmstadt, Germany) with TaqMan Universal PCR Master Mix. Primers and probes are referenced from previous studies and sequences are provided in Additional file 1: Table S1. The reaction mixture consisted of 1 × TaqMan universal PCR master mix (Life Technologies, Darmstadt, Germany), 400 nmol/L final concentrations of each primer, 200 nmol/L FAM-MGB probe, and 2 μL template for a total reaction volume of 25 μL, Thermocycler conditions were 50 °C for 2 min and 95 °C for 10 min, 40 cycles of 95 °C for 15 s, and 60 °C for 1 min. Each qPCR reaction included a negative control using nuclease-free water (Takara, Shiga, Japan) as template to exclude contamination from reagents and the environment.

### Diagnostic assessment of MinION-based mNGS.

Sensitivity and specificity of mNGS were calculated using two criteria: (1) a gold standard based on clinical culture for fungi and bacteria, and (2) a composite standard based on a combination of clinical testing (culture, microscopy, and pathology), confirmatory qPCR testing, and clinical adjudication by doctors.

The specific scoring algorithm for detecting bacterial and fungal pathogens is detailed in Table 1. Following the gold standard of conventional culture, samples displaying complete concordance with mNGS results were classified as true positives/negatives (TP/TN). Samples showing discordant results were categorized as false positives/negatives (FP/FN). Based on the composite standard, samples with inconsistent results between culture and mNGS were further assessed for the accuracy of their mNGS results by confirmatory qPCR and clinical adjudication. Those deemed accurate were scored as TP/TN, while those that did not meet these criteria were designated as FP/FN. In both standards, each sample received a maximum score of 1, and in cases where multiple microbes were identified as positive, this score of 1 was divided into fractions corresponding to the number of microbes.

**Table 1:** Specific scoring algorithm for the detection of bacterial and fungal pathogens

Gold standard or composite standard	mNGS	TP/FN score	TN/FP score
Positive for 1 organism	Positive for the identical organism	1 TP	
	Positive for different organism(s) or negative	1 FN	
Positive for 2 organisms	Positive for the 2 identical organisms	1 TP	
	Positive for only 1 identical organism	0.5 TP and 0.5 FN	
	Positive for the different organism(s) or negative	1 FN	
Positive for 3 organisms	Positive for the 3 identical organisms	1 TP	
	Positive for only 2 identical organisms	2/3 TP and 1/3 FN	
	Positive for only 1 identical organism	1/3 TP and 2/3 FN	
	Positive for the different organism(s) or negative	1 FN	
Positive for 4 organisms	Positive for the 4 identical organisms	1 TP	
	Positive for only 3 identical organisms	3/4 TP and 1/4 FN	
	Positive for only 2 identical organisms	2/4 TP and 2/4 FN	
	Positive for only 1 identical organism	1/4 TP and 3/4 FN	
	Positive for the different organism(s) or negative	1 FN	
Negative	Negative		1 TN
	Positive for any organism(s)		1 FP

TP, true positives; FN, false negatives; TN, true negatives; FP, false positives

### Statistical analysis

Normality was tested for all datasets using the D'Agostino Pearson omnibus normality test. Mann–Whitney test, Fisher’s exact test, Chi-square test or Friedman test was used as appropriate to calculate the statistical significance. A *p* value < 0.0500 indicated a significant difference. All the above statistical analysis were performed with GraphPad prism 8.

## Results

### Clinical characteristics of patients

The clinical characteristics of all the 213 suspected UTI patients in this study are presented in Table 2. Among the 213 patients, 165 were hospitalized patients, 135 of which were catheter indwelled; and with comorbidities like chronic renal disease, malignancy, and pulmonary disease etc. For the 48 outpatients, 33 were diagnosed as acute cystitis by clinical testing and 2 were nephritis; others were regarded as suspected UTI with negative microbiological results. The white blood cells count was significantly higher in the culture-positives than the culture-negatives (143.80 vs 12.60, *p* < 0.0001, Mann–Whitney test). In addition, there was no significance observed in the percentage of neutrophils (76.75 vs 71.10, *p* = 0.0559, Mann–Whitney test), C-reactive protein (18.05 vs 23.20, *p* < 0.0001, Mann–Whitney test) and procalcitonin (0.2170 vs 0.2000, *p* < 0.0001, Mann–Whitney test) between these two groups (Table 2). Among the UTI patients with culture-positive results, 78 had mono-fungal infections, 57 had mono-bacterial infections, and 13 had polymicrobial infections.

**Table 2:** Clinical characteristics of patients and laboratory findings

	Culture positive(n = 148)	Culture negative (n = 65)	*P* value
Age, median (IQR), years	75 (64–84)	62.5 (56.75–74)	** < 0.0001**^**a**^
Gender, n (%)			
Male	75 (50.68)	42 (64.62)	0.0597^b^
Female	73 (49.32)	23 (35.38)	0.0597^b^
Comorbidities, n (%)			
Diabetes	4 (2.70)	4 (6.15)	0.2225^b^
Pulmonary disease	18 (12.16)	4 (6.15)	0.1846^b^
Cardiovascular disease	5 (3.38)	3 (4.62)	0.6619^b^
Cerebrovascular disease	9 (6.08)	3 (4.62)	0.6692^b^
Malignancy	27 (18.24)	12 (18.46)	0.9697^b^
Chronic renal disease	9 (6.08)	15 (23.08)	**0.0003**^**b**^
Chronic liver disease	10 (6.76)	3 (4.62)	0.5477^b^
Haematological disease	4 (2.70)	2 (3.08)	0.8792^b^
Hospital, median (IQR), days	25.50 (15–63.5)	12.50 (7.75–25)	** < 0.0001**^**a**^
Catheterized, n (%)	106 (71.62)	29 (44.62)	**0.0002**^**b**^
WBC count, (RR, 0–30), /μL	143.80 (1.3–32,444.8)	12.60 (1–4544.9)	** < 0.0001**^**a**^
Unknown	34 (22.97)	1 (1.54)	** < 0.0001**^**c**^
0–30	22 (14.87)	42 (64.61)	
> 30	92 (62.16)	22 (33.85)	
PON, (RR, 40–75)	76.75 (1.1–95.3)	71.10 (30.9–95.8)	0.0944^a^
Unknown	38 (25.67)	2 (3.08)	** < 0.0500**^**c**^
< 40%	3 (2.03)	1 (1.54)	
40–75%	49 (33.11)	40 (61.54)	
> 75%	58 (39.19)	22 (33.84)	
CRP, (RR, < 10), mg/L	18.05 (0.04–232.8)	23.20 (0.5–275.9)	0.2971^a^
Unknown	43 (29.05)	4 (6.15)	> 0.0500^c^
< 10	42 (28.38)	24 (36.92)	
10–50	31 (20.95)	14 (21.54)	
> 50	32 (21.62)	23 (35.39)	
PCT, (RR, < 0.05), ng/mL	0.2170 (0.02–14.95)	0.2000 (0.02–17.11)	0.4738^a^
Unknown	64 (43.24)	38 (58.46)	> 0.0500^c^
< 0.05	11 (7.43)	4 (6.15)	
> 0.05	73 (49.33)	23 (35.39)	

Bolded *p*-values indicate significant values (*p* < 0.0500)

IQR, interquartile range; WBC, white blood cells; PON, percentage of neutrophils; CRP, C-reactive protein; PCT, procalcitonin; RR, reference range

^a^significance was determined by Mann–Whitney test

^b^significance was determined by Chi-square test

^c^significance was determined by Fisher’s exact test

### Diagnostic performance of MinION-based mNGS

**Fig. 2:**
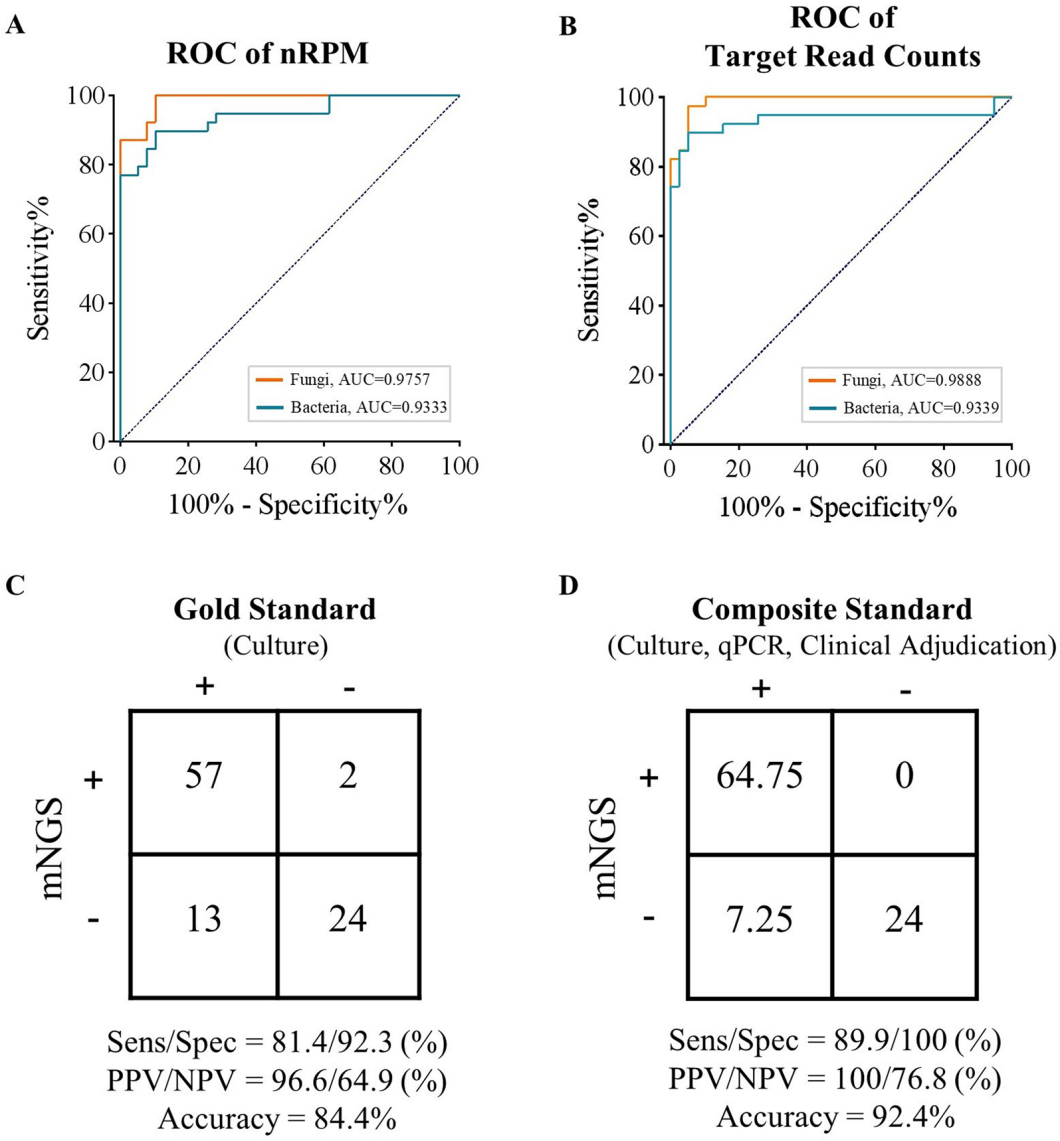
Accuracy evaluation of mNGS testing. **A**, **B** ROC curve of fungal and bacterial training datasets based on culture results. **C**, **D** Contingency tables for the independent validation dataset based on the gold standard culture and composite standard, respectively. Sens, sensitivity; Spec, specificity; PPV, positive predictive value; NPV, negative predictive value

The nRPM threshold was set to 0.01 with an AUC value of 0.9757 for fungi, and 0.275 with an AUC value of 0.9333 for bacteria, respectively (Fig. 2A), based on the optimal Youden’s index derived from the training dataset ROC curve. The lowest-read number threshold of target pathogen(s) was set to 5 with an AUC value of 0.9888 for fungi and 153 with an AUC value of 0.9339 for bacteria, respectively (Fig. 2B). Detailed information of bacterial and fungal pathogens detection results is reported in Additional file 2: Table S2 and the raw data of the culture-negatives (negative control) is shown in Additional file 3: Table S3, Additional file 4: Table S4.

#### Based on gold standard

In the validation dataset, out of the 70 samples with positive culture results, 58 (82.9%) were also identified as positive by mNGS testing, and pathogens were completely consistent in 43 (61.4%) of these cases. The remaining 15 samples exhibited partial concordance, suggesting the presence of at least one overlap of pathogens, especially when polymicrobial results were observed. Among the 12 false-negative (fully inconsistent) samples, 8 (66.7%) displayed different positive results and 4 (33.3%) had no microbes met the positive diagnostic criteria of mNGS. Finally, mNGS testing scored 57 out of 70 and gave a final sensitivity of 81.4% (95% confidence interval (CI) 70.64–88.95%) with a positive predictive value (PPV) of 96.6% (95% CI 87.78–99.74%) based on the gold standard culture testing (Fig. 2C). For the 26 culture-negative samples, only two (CN41 and CN50) had positive pathogens results, resulting in a specificity of 92.3% (95% CI 74.74–98.98%) and negative predictive value (NPV) of 64.9% (95% CI 48.7–78.23%). We also analyzed the mNGS results of the 39 samples from healthy individuals and identified that 4 of them (H17, H21, H33, H35) met the positive detection criteria. This suggests the presence of asymptomatic bacteriuria (ASB) in the healthy cohort, albeit at a lower proportion.

#### Based on composite standard

After adjudication by the composite standard, the mNGS results were confirmed for the 8/12 fully unconcordant culture-positive samples (CP44, CP63, CP79, CP135, CP139, CP141, CP142, CP145) which elevated the scores by 5.75. Of the entire validation dataset, only 10 pathogens were missed in all by mNGS; of these, 8 were presented in the mNGS results but were excluded for failure to meet all three positive criteria. For the FP samples (CN41 and CN50) based on gold standard, the diagnosis results of the mNGS workflow were further confirmed credible by qPCR testing and clinical adjudication. Therefore, the sensitivity and specificity were 89.9% (95% CI 85.87–92.94%) and 100% (95% CI 83.69–100%), respectively (Fig. 2D), and the PPV and NPV were 100% (95% CI 98.25–100%) and 76.8% (95% CI 68.63–83.38%), respectively.

#### Detection of DNA viruses

Of the 65 culture-negative samples, 22 were regarded as positives by mNGS testing and mostly (21, 95.5%) were JC polyomavirus (JCV). The remaining 1 (0.5%) were positive for BK polyomavirus (BKV). To further validate the mNGS results above, qPCR testing was performed on all the samples that were positive for DNA virus by mNGS. As expected, all 22 samples showed positive qPCR results in line with the mNGS testing. We also investigated DNA viruses in 148 culture-positive samples and found that in only one sample (CP42) were reads from a virus (JCV) detected and validated by qPCR (see Additional file 5: Table S5 for detailed information).

### Potential of point-of-care testing (POCT) by MinION-based mNGS

#### Improved diagnostic performance of mNGS testing compared to culture testing

**Fig. 3:**
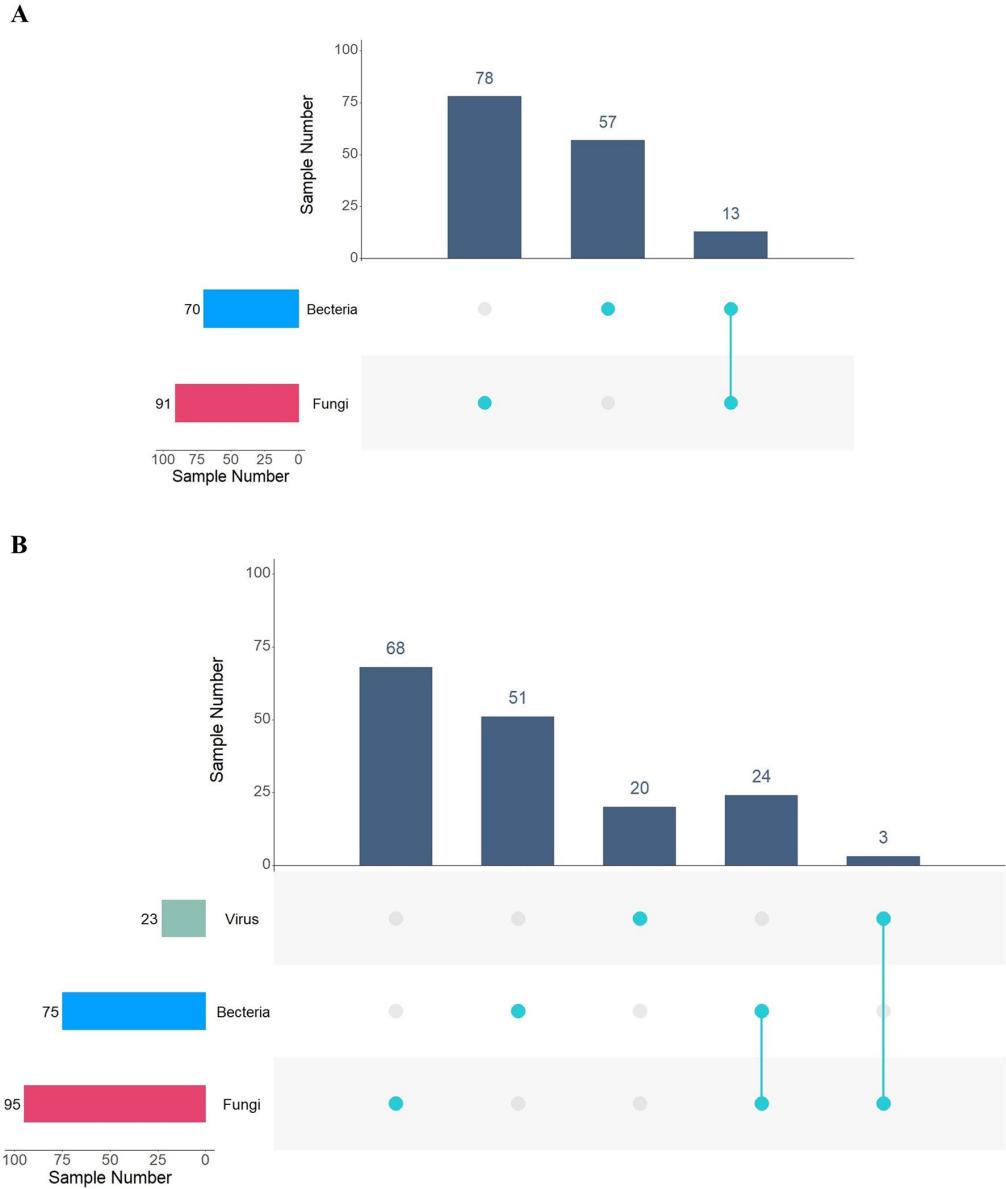
Comparison analysis of culture and mNGS testing for pathogen distribution and composition. **A** The culture-based results. **B** The mNGS-based results. The horizontal axis and vertical axes represent number of samples in the interacting sets of one or multiple pathogenic infection types and number of samples in each pathogenic infection type, respectively

Comparison of the results of the culture with the MinION-based mNGS testing showed that culture testing detected bacterial pathogens in 70 samples and fungal pathogens in 91 samples (Fig. 3A). In contrast, mNGS detected bacterial pathogens in 75 samples and fungal pathogens in 95 samples (Fig. 3B), showing a comprehensive improvement in the diagnostics of infections caused by not only bacterial but fungal pathogens. This was also a great improve in terms of the detection of polymicrobial infections: 13 polymicrobial samples were detected by culture and 29 samples were detected by mNGS. Importantly, this mNGS-based method can detect dsDNA viruses which are not detectable by culture in clinics, and further found 3 polymicrobial infected samples related to viruses (Fig. 3B).

#### APDNS (Automatic pathogen diagnosis by nanopore sequencing) for automated pathogen identification

The availability of rapid and simple bioinformatic analysis that can be used in real time is a critical requirement for the widespread use of mNGS in clinical microbiology. To enable rapid analysis of MinION-based mNGS data and output a definite pathogen identification result directly, bioinformatics analysis and positive threshold were combined into an automatic pathogen identification pipeline named APDNS, which allows the base-called data as the input file and directly outputs a csv file including species name, nRPM value, and read number of suspected pathogens (see Additional file 6: Table S6 for an example of the output result from APDNS). This allows for a clear detection result with no further calculations required. APDNS was tested using a few randomly selected samples and found that a ~ 5 min turnaround time after sequences acquisition could identify pathogens successfully in some samples such as CP18 and CP31.

#### mNGS based on MinION enabled significant reduction of the turnaround time

**Fig. 4:**
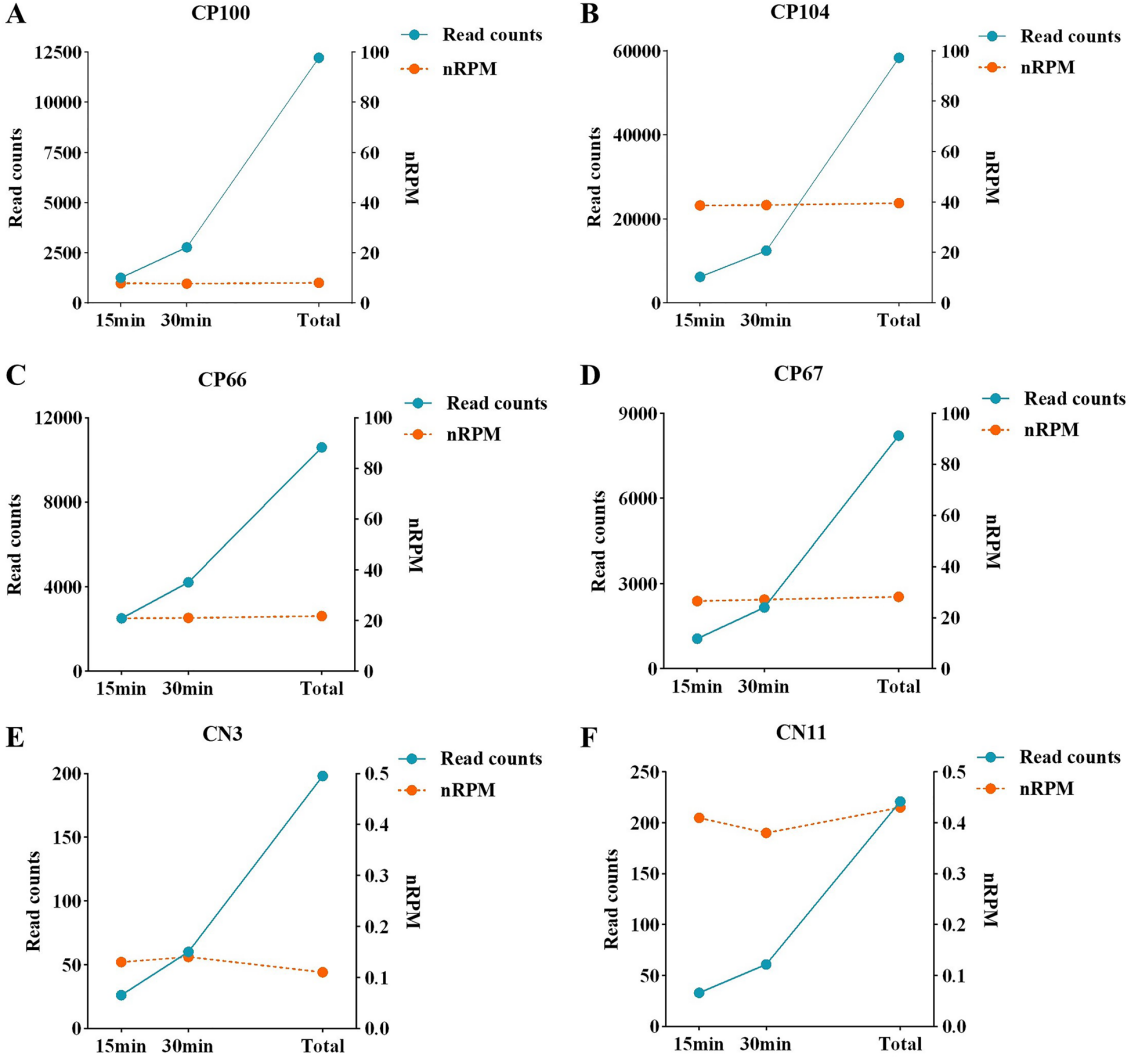
Detection of the target reads over time. The line charts represent target read counts and RPM value at times 15 min, 30 min, and total time, respectively. **A** and **B** are from bacterial samples. **C** and **D** are from fungal samples. **E** and **F** were viral samples. The title of each graph corresponds to the sample number

MinION sequencing enabled real-time analysis of the output reads so that it has the great potential to further reduce the turnaround time by shortening the sequencing time. A timepoint (15 min, 30 min, and total time) analysis for sequencing data of all the 163 mNGS-positive samples was performed to evaluate if a shorter sequencing time would be sufficient for pathogen identification (Additional file 7: Table S7). The results showed that the same pathogens could be detected successfully at 15 min, except 4 fungal-positive samples (CP37, CP53, CP76, CP136), indicating that ≤ 15 min of sequencing time would be sufficient for pathogen identification in most cases. The nRPM was found to not change significantly (3.12 vs 3.32 vs 3.30, *p* > 0.0500, Friedman test) as the reads number increased (189 vs 398 vs 1076, *p* < 0.0001, Friedman test). 6 samples that were detected as positive for a pathogen—2 bacterial (CP100 and CP104, Fig. 4A, B), 2 fungal (CP66 and CP67, Fig. 4C, D), and 2 viral (CN3 and CN11, Fig. 4E, F) pathogens were randomly selected and visualized in Fig. 4.

## Discussion

The use of mNGS for pathogen diagnosis in infectious diseases is not a new concept. Several studies have demonstrated the significant utility of mNGS in pathogen detection of relatively sterile human body environments, such as the central nervous system and cardiovascular system, and have established applicable diagnosis standards [4, 20, 23, 24]. Nevertheless, the human body hosts trillions of microbes, which complicates the establishment of pathogen detection standards for specimens from nonsterile environments such as the urinary system. mNGS protocols for such systems have not been constructed and evaluated comprehensively. Urine can be collected non-invasively in large quantities, making it an appealing target for diagnostic assays. In this study, we developed a MinION-based mNGS diagnostic approach to enhance pathogen detection capabilities and ensure that background DNA was disregarded while authentic pathogens were accurately identified. We further assessed its performance in diagnosing pathogens in UTI patients using clinical urine samples.

The MinION is an attractive mNGS platform for diagnosing infectious diseases because of its rapid diagnosis capability. Through comparative analysis of the numerous identified pathogens, it was confirmed that MinION-based mNGS testing outperformed conventional culture. Based on the final composite standard, the diagnosis accuracy was notably increased from 84.4 to 92.4% using mNGS testing compared to the culture method. The negative predictive value (76.8%) was slightly lower since there were fewer negative samples in the validation dataset, but this increased to 84.0% when calculated in the total sample dataset. Furthermore, the mNGS testing increased the possibility of detecting all the potential pathogens presented including bacteria, fungi, and dsDNA viruses, while the conventional culture method could not identify currently unculturable microorganisms. These discrepancies highlight the utility of using novel mNGS testing to overcome deficiencies of conventional culture. The high detection rate of viruses in culture-negatives is a reminder that culture-negative samples deserve more attention and need a further diagnosis by another detection method like mNGS testing.

Due to the widespread adoption of new immunosuppressive and immunomodulatory treatments, there has been a significant rise in the risk of microbiome dysbiosis and an increase in the number of immunocompromised patients. Consequently, it is not surprising to observe an uptick in the detection of rare pathogens, including viruses and complex polymicrobial infections [25]. Compared to the clinical gold standard of in vitro culturing, more samples were identified as polymicrobial infections by mNGS testing, showing the great advantage of mNGS testing in the diagnosis of pathogens in complicated polymicrobial infections. In addition, the result obtained by this study also indicates that the clinicians need to be more aware of the possibilities of highly ubiquitous and usually harmless DNA viruses such as BKV and JCV serving as causal agents of UT diseases, especially among immunocompromised patients and transplant recipients [26–28].

The 4 samples (H17, H21, H33, H35) from healthy individuals which reached the positive criteria were regarded as ASB by clinician, a condition that not merits treatment generally [29]. As expected, these 4 samples also detected of probiotics (*Lactobacillus crispatus* or *Lactobacillus iners*), though the top dominant reads belonging to pathogens. In the contrast, we rechecked the mNGS results from patients with suspected UTI and no probiotics was observed in most (207/213, 97.2%) samples of them. In other words, the existence of probiotics appears to establish a microenvironment that shields the host from uropathogenic microbe infections, as elucidated in a previous study [30]. The presence of ASB among the healthy individuals also serves as a reminder that the diagnosis of UTIs based on mNGS must be complemented by clinical factors such as gender, age, and symptoms.

Most clinical diagnosis assays tend to be converted to a real-time format that will reduce hands-on laboratory time and effort, in turn, decreases the overall turnaround time from sample collection to obtaining results [23]. The mNGS platform presented in this study has the great potential to achieve this goal and advance a mNGS-based POCT implementation. The custom bioinformatics pipeline developed here can greatly ease the demand for bioinformatics skills and improve the speed of data analysis. Importantly, in all cases from this study, sequencing for ≤ 1 h was sufficient for pathogens identification. The target pathogen reads analysis according to sequencing time indicated that a 15-min sequencing run was adequate for pathogen detection in some cases. The entire turnaround time for pathogen identification by mNGS testing (including 1.5 h of DNA extraction, 2 h of library preparation, 15 min of sequencing, and 5 min of bioinformatic analysis) in this study can be reduced to about 4 h, which is much faster than standard urine culture testing, which usually requires ≥ 24 h. With the additional advantage of the small size of the MinION sequencer, it has the greater potential for pathogen detection on site.

Many limitations of this study should be recognized. An important limitation is that the mNGS workflow is still not truly comprehensive since RNA viruses were not included. Additionally, the mNGS-based virus-positive samples were not validated by a third assay, because in these samples qPCR and mNGS testing gave fully consistent results. The cost (~ $92 per sample) of nanopore sequencing and the materials used in this study were still high, though reduction of the cost was attempted by sequencing 6 samples per run and reusing the flow cell several times, at the base of avoiding cross-over contamination. The diagnostic workflow developed in this study for identification of potential pathogens relied on reference databases of previously sequenced organisms. This confined detection and identification to species previously identified and annotated. As a result, false negatives may occur due to incomplete or missing taxonomic representation in databases.

In this study, we developed a MinION-based mNGS workflow designed to accurately differentiate pathogens from the numerous normal microbes present in UTIs. Of particular significance, the automated pathogen identification pipeline developed in this study was capable of delivering precise pathogen identification within approximately 5 min of obtaining sequences. As the sequencing library preparation workflow continuously improves, it is anticipated that the MinION-based mNGS workflow presented in this study will be applied to the clinical practice of POCT in pathogen diagnosis of UTIs and other infectious diseases. In conclusion, this study demonstrated that by combining unbiased organism lysis, untargeted sequencing, auto-rapid data analysis, and comprehensive reference databases, mNGS can be applied in real clinical practices for hypothesis-free, universal pathogen detection, promising to improve diagnostic yield for syndromic testing of all microbiological infections.

### Supplementary Information

**Additional file 1: Table S1. **Primers and probes used for confirmatory qPCR.

**Additional file 2: Table S2. **Detailed information of bacterial and fungal pathogens detection result by mNGS testing.

**Additional file 3: Table S3. **Reads information of top 1 bacterial species detected by mNGS testing in training culture-negative samples.

**Additional file 4: Table S4. **Reads information of top 1 fungal species detected by mNGS testing in training culture-negative samples.

**Additional file 5: Table S5. **mNGS Results for Detecting Virus.

**Additional file 6: Table S6. **Example of the output result from the data processing pipeline APDNS.

**Additional file 7: Table S7. **Timepoint analysis of nRPM and read counts for the mNGS-positive samples at 15min, 30min, and total time.

## Data Availability

The trimmed MinION sequencing reads can be accessed in the NCBI Sequence Read Archives (SRA) under BioProject accession number PRJNA854064.
